# Facial follicular porokeratosis simulating discoid lupus erythematosus in an African American woman

**DOI:** 10.1016/j.jdcr.2025.02.031

**Published:** 2025-03-14

**Authors:** Pritika Singh, Layna Mager, Jeffrey Y. Liu, Jason B. Lee, Sylvia Hsu

**Affiliations:** a Pennsylvania State University College of Medicine, Hershey, Pennsylvania; b The Ohio State University College of Medicine, Columbus, Ohio; c Department of Dermatology, Temple University Lewis Katz School of Medicine, Philadelphia, Pennsylvania; d Department of Dermatology and Cutaneous Biology, Sidney Kimmel Medical College at Thomas Jefferson University, Philadelphia, Pennsylvania

**Keywords:** dermatopathology, discoid lupus erythematosus, porokeratosis

## Introduction

Porokeratosis is a disorder of abnormal keratinization that often presents with keratotic papules to annular plaques with elevated borders. The lesions typically occur on sun-exposed skin with most presenting in the fifth decade of life but can occur at any age.^1^ Lesions are most often distributed on the extremities and trunk. Though facial involvement can be seen in up to 15% of patients with disseminated superficial actinic porokeratosis, single, solitary lesions are rare.^2^, ^3^, ^4^ Follicular involvement of porokeratosis is infrequently seen, though it has been reported in cases of various variants of porokeratosis.^5^, ^6^, ^7^, ^8^, ^9^ In this report, we highlight a case of a single, solitary, atrophic lesion on the face of a 32-year-old African American female patient that was previously diagnosed and treated as discoid lupus erythematosus (DLE) with histology showing cornoid lamella within the follicle.

## Case presentation

**Fig 1:**
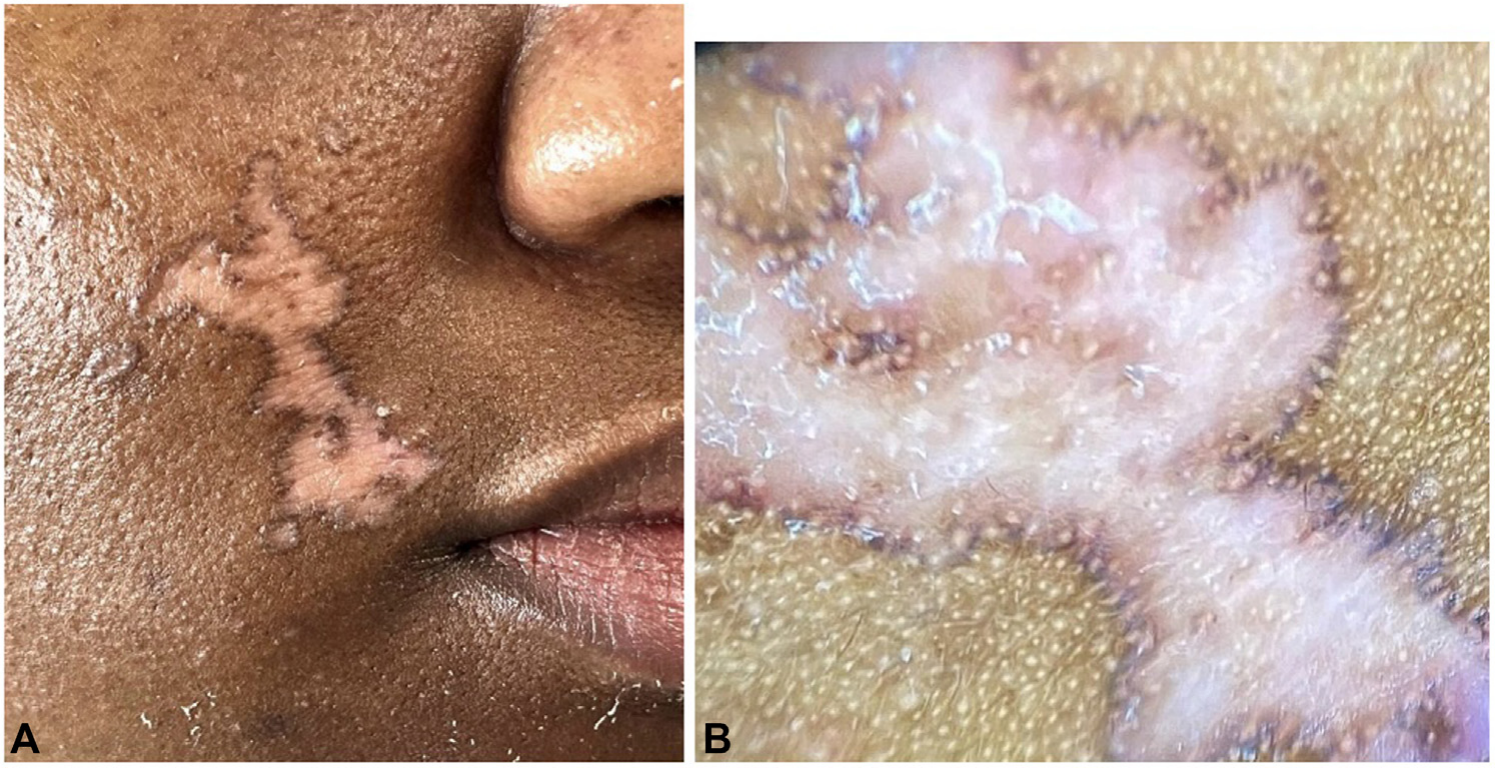
**A,** Atrophic patch with central hypopigmentation and a brown raised border on the right medial cheek. **B,** Same patch under 4× magnification.

An African American woman in her 30s with no significant past medical history presented to our outpatient clinic with concern for a lesion on her right cheek. The patient reports that the lesion was present for 5 years with no associated symptoms. This lesion was previously diagnosed as DLE by a rheumatologist and was started on hydroxychloroquine at that time. Serology was negative for antinuclear, antidouble-stranded DNA, anticentromere, anti-Smith/ribonucleoproteins, anti-Sjogren antibodies anti-Sjögren's syndrome A (Ro) and anti-Sjögren's syndrome B (La) antibodies, anti-Jo1, anticardiolipin, and antibeta-2 glycoprotein 1 antibodies. Complement levels were normal. She was referred to dermatology for further management. On clinical examination, the patient presented with an atrophic patch with central hypopigmentation and a brown raised border on the right medial cheek (Fig 1, *A* and *B*).

**Fig 2:**
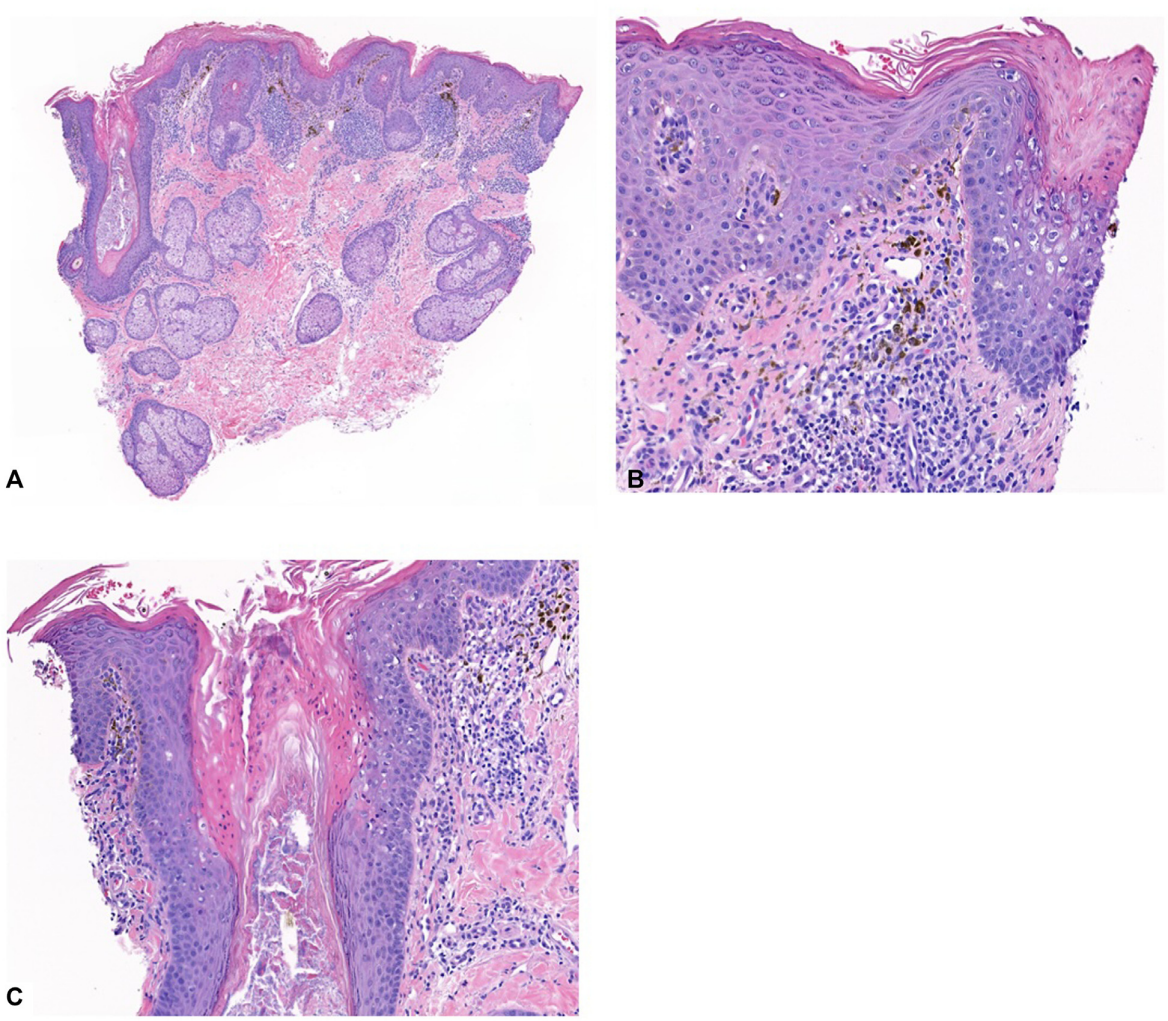
**A,** Moderately dense superficial perivascular lymphocytes and melanophages without active vacuolar interface changes of the epidermis and infundibula. **B** and **C,** In 2 of the 4 follicles, multiple dyskeratotic cells were seen within the infundibula. In the other 2, there were characteristic histopathologic changes of follicular porokeratosis: a column of parakeratosis with dyskeratotic cells surrounded by vacuolated and pale epithelial cells.

A 3-mm punch biopsy of the raised border showed moderately dense superficial perivascular lymphocytes and melanophages without active vacuolar interface changes of the epidermis and infundibula (Fig 2, *A*). In 2 of the 4 follicles, multiple dyskeratotic cells were seen within the infundibula. In the other 2, there were characteristic histopathologic changes of follicular porokeratosis: a column of parakeratosis with dyskeratotic cells surrounded by vacuolated and pale epithelial cells (Fig 2, *B* and *C*). With the histopathologic findings, the patient was given the diagnosis of porokeratosis and advised to discontinue hydroxychloroquine. The patient was subsequently treated with 2% topical lovastatin twice daily in the affected area, with a planned follow-up 3 months after the visit.

## Discussion

Porokeratosis often presents as keratotic papules or annular plaques that expand centrifugally with an elevated keratotic border. Centrally, the lesion can appear slightly atrophic with raised borders. Lesions are more frequently distributed to photo-exposed areas, including the extremities, trunk, and face. The etiology and pathology of porokeratosis is unclear. UV light exposure, as well as repeated frictional trauma, has been suggested.^1^ There are multiple clinical variants of porokeratosis that can look clinically distinct from one another, but all share the classic histological finding of cornoid lamella, a column of compact parakeratosis with a focal absence of the granular layer, and the concomitant presence of dyskeratotic cells.^2^^,^^10^

Follicular porokeratosis is suggested to be a histopathological variant of porokeratosis with cornoid lamella restricted to follicles.^4^^,^^5^ Clinically, follicular porokeratosis can present with isolated facial involvement. DLE can have a similar clinical presentation with most lesions also localized to the face, ears, and scalp. Lesions of DLE may also exhibit a photodistribution, as sun exposure may also play a role in the development of lesions.^6^ DLE lesions appear as well-defined, annular erythematous patches or plaques of varying sizes followed by follicular hyperkeratosis. These lesions slowly expand with active inflammation and hyperpigmentation at the periphery, leaving depressed central atrophy, scarring, telangiectasia, and hypopigmentation, which can simulate the atrophic lesions of porokeratosis on the face.^7^^,^^8^

Histopathologic findings can also be similar in both entities, as follicular porokeratosis can simulate follicular plugging seen in DLE. The latter consists of compact orthokeratosis that can lead to columns of parakeratosis if the interface dermatitis is brisk around the infundibulum. These findings are indistinguishable from cornoid lamella in follicular porokeratosis.^8^ However, follicular porokeratosis usually lacks vacuolar interface changes involving the infundibulum. Another distinguishing histopathologic finding of porokeratosis is the presence of vacuolated or pale infundibular epithelial cells, which were observed in our case.^9^ Of the few cases reported, one was reported in a White patient that was initially treated for DLE with hydroxychloroquine.^7^ Only one other case was reported in an African American patient.^8^

Without medical or surgical intervention, porokeratosis will remain indefinitely. Spontaneous regression of these lesions is extremely rare. Treatments with variable results have been used for porokeratosis, including topical 5-fluorouracil, keratolytics, retinoids, vitamin D-3 analogs, and excision. There are no international guidelines on treatment standards. However, marginal improvements in lesion size have been reported with the use of topical lovastatin.^1^

## Conclusions

Our case presents a rare clinical subtype of porokeratosis with follicular involvement on the face of an African American woman. This entity simulates DLE and is rarely reported in skin of color patients. Taken together, it is important for dermatologists to recognize this presentation to avoid misdiagnosis and unnecessary treatment. Histopathological evaluation is necessary to establish a definitive diagnosis.

## Conflicts of interest

None disclosed.
